# Daily urinary creatinine predicts the weaning of renal replacement therapy in ICU acute kidney injury patients

**DOI:** 10.1186/s13613-016-0176-y

**Published:** 2016-07-22

**Authors:** Nicolas Viallet, Vincent Brunot, Nils Kuster, Delphine Daubin, Noémie Besnard, Laura Platon, Aurèle Buzançais, Romaric Larcher, Olivier Jonquet, Kada Klouche

**Affiliations:** Department of Intensive Care Medicine, Lapeyronie University Hospital, 371, Avenue du Doyen G. Giraud, 34295 Montpellier, France; Department of Biochemistry, Lapeyronie University Hospital, Montpellier, France; PhyMedExp, INSERM U1046, CNRS UMR 9214, University of Montpellier, Montpellier, France

**Keywords:** Acute kidney injury, Renal replacement therapy, Weaning, Urine output, Urinary creatinine

## Abstract

**Background:**

In acute kidney injury (AKI), useless continuation of renal replacement therapy (RRT) may delay renal recovery and impair patient’s outcome. In this study, we aimed to identify predictive parameters that may help to a successful RRT weaning for AKI patients.

**Methods:**

We studied 54 surviving AKI patients in which a weaning of RRT was attempted. On the day of weaning (D_0_) and the following 2 days (D_1_ and D_2_), SAPS II and SOFA scores, 24-h diuresis, 24-h urinary creatinine and urea (UCr and UUr), creatinine and urea generation rates (CrGR and UrGR) and clearances (CrCl and UrCl) were collected. Patients who remained free of RRT 15 days after its discontinuation were considered as successfully weaned.

**Results:**

Twenty-six RRT weaning attempts succeeded (S_+_) and 28 failed (S_−_). Age, previous renal function, SAPS II and SOFA scores were comparable between groups. At D_0_, 24-h diuresis was 2300 versus 1950 ml in S_+_ and S_−,_ respectively, *p* = 0.05. At D_0_, D_1_ and D_2_, 24-h UUr and UCr levels, UrCl and CrCl, and UUr/UrGR and UCr/CrGR ratios were significantly higher in S_+_ group. By multivariate analysis, D_1_ 24-h UCr was the most powerful parameter that was associated with RRT weaning success with an area under the ROC curve of 0.86 [0.75–0.97] and an odds ratio of 2.01 [1.27–3.18], *p* = 0.003.

**Conclusions:**

In ICU AKI, 24-h UCr appeared as an efficient and independent marker of a successful weaning of RRT. A 24-h UCr ≥5.2 mmol was associated with a successful weaning in 84 % of patients.

## Background

Acute kidney injury (AKI) is a common condition in the intensive care unit (ICU). Approximately 5 % of ICU patients suffering from AKI require renal replacement therapy (RRT) [1]. Such patients have a hospital mortality rate of 45–70 % [1–6], and those surviving to hospital discharge continue to carry a high risk for long-term morbidity and mortality [7–10]. Several studies have shown that between 10 and 30 % of patients with AKI who required RRT will remain dialysis dependent at discharge [7–13]. Yet, renal function in AKI surviving patients requiring RRT ultimately recovers in the vast majority of case (more than 90 % of patients) [14]. However, performances of RRT may have untoward effects that contribute to the prolongation of renal failure or impede the ultimate recovery of renal function [15]. Indeed, RRT represents an independent and an added risk factor for mortality [16–18] as it exposes the patient to several complications like catheter-related infections or thrombosis, bleeding favored by anticoagulation, hemodynamic instability. The prolonged continuation of RRT could also be deleterious for renal recovery, particularly in cases of intradialytic hemodynamic instability, inducing renal ischemic lesions [19]. Renal biopsy in patients with prolonged AKI treated by hemodialysis showed regions of fresh tubular necrosis days-to-weeks after the initial insult [20]. RRT may also delay renal recovery possibly by purification of mediators and growth factors needful for the tubular repair [21]. Moreover, RRT may remove unpredictable amounts of antibiotics, amino acids and nutrients. Also, RRT represents a significant additional cost in the patient’s care. It is therefore a matter of concern to identify which factors present at the time of discontinuation may help physicians in predicting successful cessation of RRT [22]. To the best of our knowledge, a paucity of studies has been reported on RRT weaning during AKI [23–26]. Sepsis-related Organ Failure Assessment (SOFA) score [23, 26] and 2-h creatinine clearance [25] have been reported as a reliable predictive factors, but urine output remains the most consistent parameter significantly associated with the success of weaning [24]. This study was therefore undertaken to investigate and identify parameters, particularly 24-h urinary creatinine, present at the time of RRT weaning that would be associated with successful cessation of RRT during AKI.

## Patients and methods

This retrospective study was conducted, from January 2008 to December 2012, in two medical ICUs of the Montpellier University Hospital, France. It was approved by our local institutional review board (Ethic Committee of Montpellier; Comité de protection des personnes: CPP Sud Mediterranée 4) which waived informed consent from the patients or their relatives.

### Study population

Patients admitted to the ICU for AKI requiring RRT that survived and in which an attempt of weaning was realized were enrolled in the study. Exclusion criteria included pregnancy, age <18 years old, previous chronic renal failure stage ≥4, AKI of obstructive, glomerular or vascular etiology. AKI patients that underwent acute RRT for less than 3 days (no attempt of weaning), and patients without an attempt of weaning RRT or transferred to another unit before an attempt of weaning were also excluded. Decisions regarding the initiation, management and discontinuation of RRT were made by the referring physician according to the guidelines of the intensive care and nephrology societies [22]. The choice of RRT modality was depending on patient hemodynamic stability and was daily re-evaluated. Patients with hemodynamic instability or severe fluid overload were preferentially treated with pre-dilutional continuous venovenous hemodiafiltration (CVVHDF) or on-line sustained low-efficiency daily dialysis-filtration (SLEDD-f), and those with hemodynamic stability were preferentially treated with on-line intermittent hemodiafiltration (IHDF) [27].

### Study design

An RRT cessation was considered as an attempt of weaning when referred to a medical decision and if it lasted more than 72 h. Only the first attempt was analyzed for each patient. Current criteria required for weaning attempt were the association of an urine output of at least 20 ml/h without diuretics [24, 28], a restored and stable hemodynamic and respiratory conditions, no new kidney aggression (mainly toxic and predictable), no need to continue RRT for the previously mentioned reasons. A successful weaning was defined as the cessation of RRT for at least 15 days. This endpoint defined two groups of patients: S_+_ for the success of the weaning attempt and S_−_ if it failed.

### Data collection

Baseline patient characteristics were recorded, including age, gender, cause of AKI, comorbidities, previous renal function (estimation of glomerular filtration rate—eGFR, by the Chronic Kidney Disease Epidemiology, CKD-epi formula [29]), weight, urine volume. Severity of illness was determined at the inclusion using the simplified acute physiology score II (SAPS II) and the Sequential Organ Failure Assessment (SOFA) score. RRT modalities were collected during treatment.

Day 0 (D_0_) was defined as the day of RRT cessation at the weaning attempt, and D_1_, D_2_ the 2 following days. From D_0_ to D_2_, we daily collected the following parameters: non-renal SOFA score, patient’s weight, 24-h urine output, dose of furosemide, fluid balance (intake output), mean arterial pressure, dose of vasopressors, use of mechanical ventilation, creatinine and urea blood and urine levels. Blood samples were drawn and 24-h urine samples were collected at D_0_ and every following day. Creatinine and urea clearance (CrCl and UrCl), diuretic response index and creatinine and urea generation rate (CrGR and UrGR, respectively) were estimated at D_1_ and D_2_ according to:

- Diuretic response index (ml/mg) = 24-h urine output (ml)/daily dose of furosemide (mg)
- CrGR D_1_ (µmol/mn) = [(SCrD_1_ − SCrD_0_) × 0.6 × weight) + (UCr × urine volume)]/time D_0_ − D_1_
- UrGR D_1_ (µmol/mn) = [(SUr D_1_ − SUr D_0_) × 0.6 × weight) + (UUr × urine volume)]/time D_0_ − D_1_
- CrCl (ml/mn) = (UCr × 24-h urine volume)/(Scr × 1440)
- UrCl (ml/mn) = (UUr × 24-h urine volume)/(Sur × 1440)

where SCr: serum creatinine (µmol/l), UCr: urinary creatinine (mmol/l), SUr: serum urea (mmol/l), UUr: urinary urea (mmol/l), weight (kg), urine volume (l), time (min).

Early re-renal replacement therapy, defined as relapsed need for RRT within 15 days after D_0_ was considered as the primary outcome variable. Duration of mechanical ventilation, vasopressor treatment and RRT, hospital and 3-month mortality, and the need of RRT after 3 months were also collected.

### Statistical analyses

Statistical analyses were performed using the software R 3.1.0 (R Foundation for Statistical Computing, Vienna, Austria). We first performed a descriptive analysis by computing the frequencies and the percents for categorial data; means or medians, standard deviations, quartiles and extreme values for continuous data. We also checked for the normality of the continuous data distribution using the Shapiro–Wilk tests. The univariate analysis was performed using two-tailed Student’s *t* test for continuous variables, Fisher and Chi-square tests for categorial variables or two-tailed Mann–Whitney–Wilcoxon test when appropriate. The areas under the receiver operator characteristic (ROC) curves and their confidence interval (CI) were calculated using the method of DeLong. The optimal threshold value was set for each ROC curve through the Youden index (corresponding to the maximum of the sum sensitivity + specificity). CIs for sensitivity (Se), specificity (Sp), positive predictive value (PPV) and negative predictive value (NPV) were calculated by bootstrap (2000 bootstrap samples). The most relevant variables including urine output, serum creatinine, creatinine clearance, urea and creatinine generation rate and 24-h urinary urea and creatinine obtained by logistic regression were included in multivariate models. The urea and creatinine kinetics in the two groups of patients were modeled using mixed models. A *p* value less than 0.05 was considered statistically significant.

## Results

### Population

**Fig. 1:**
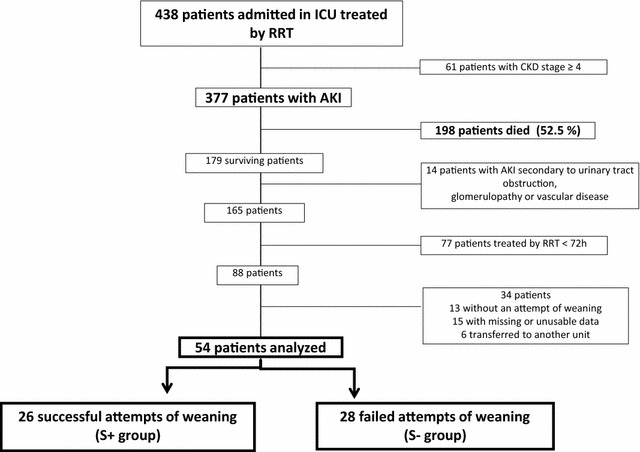
Flowchart of the study population

During the study period, 377 consecutive patients were admitted in ICUs with AKI treated by RRT in which 179 survived (survival rate 47.4 %). After exclusion of 125 patients, 54 were eligible for analysis: 18 females and 36 males (flowchart, Fig. 1). Patient’s characteristics, main comorbidities and reasons of ICU admission are summarized in Table 1. The RRT modalities were equally represented in the study population. At D_O_, non-renal SOFA score has decreased reaching a median value of 3, and more than half of population was still on mechanical ventilation (Table 2). Median duration of RRT prior to the first attempt of weaning was 11 days.

**Table 1:** Patients characteristics, comorbidities, causes of ICU admission, RRT modalities at initiation of therapy and outcome according to the success or failure of RRT weaning attempt

	All patients (*n* = 54)	S_+_ group (*n* = 26)	S_−_ group (*n* = 28)	*p*
Age (years)	63.5 [56–72.8]	61.5 [56–71]	67.5 [56.5–75.5]	0.12
Male [*n* (%)]	36 (67)	20 (77)	16 (51)	0.09
SAPS II	55 [39–69]	54 [39.5–65.8]	56 [40.5–74.5]	0.36
SOFA	11 [8–13]	11 [8.3–13]	11 [8.5–13]	0.51
Previous eGFR (ml/min/1.73 m^2^)	70 [45–92]	77 [45–96]	65 [46–89]	0.46
Main comorbidities [*n* (%)]				
Hypertension	28 (52)	12 (46)	16 (57)	0.16
Diabetes	19 (35)	8 (31)	11 (39)	
Cardiac failure	8 (15)	4 (15)	4 (14)	
Hemopathy	8 (15)	2 (8)	6 (21)	
Main reason for ICU admission [*n* (%)]				
Septic shock	28 (52)	11 (42)	17 (61)	0.15
Cardiogenic shock/cardiac arrest	10 (19)	5 (19)	5 (18)	
Post-surgical	6 (13)	3 (12)	3 (11)	
Other	10 (13)	5 (19)	5 (18)	
Initial RRT technique [*n* (%)]				
IHDF	19 (35)	6 (23)	13 (46)	0.07
CVVHDF	19 (35)	13 (50)	6 (21)	
SLEDD-f	16 (30)	7 (27)	9 (32)	
Length of ICU stay (days)	19 [8.3–42]	13.5 [8.3–32.3]	24.5 [17.5–42]	*0.012*
Duration of RRT (days)	15 [5–35]	10.5 [5–14.8]	18 [10–35]	*0.006*
In-hospitality mortality [*n* (%)]	0 (0)	0 (0)	0 (0)	–
3-month mortality [*n* (%)]	4 (7.4)	0 (0)	4 (14)	0.11
eGFR at 3 months (ml/min/1.73 m^2^)	58 [49–92]	66 [68–92]	55 [49–77]	0.08

Italic values indicate significance of *p* value (*p* <0.05)

Results are displayed in median [interquartile range] if quantitative variable; and number and percentage if categorial variable

*p*: differences between S_+_ and S_−_ groups

*SAPS II* Simplified Acute Physiology Score, *SOFA* Sequential Organ Failure Assessment, *eGFR* estimated glomerular filtration rate, *IHDF* intermittent hemodiafiltration, *CVVHDF* continuous venovenous hemodiafiltration, *SLEDD*-*f* sustained low-efficiency daily dialysis-filtration, *RRT* renal replacement therapy

**Table 2:** Characteristics of the 54 analyzed patients at the first day (D_0_) of weaning of RRT

	All patients (*n* = 54)	S_+_ group (*n* = 26)	S_−_ group (*n* = 28)	*p*
Non-renal SOFA	3 [1–6]	3 [1–5]	3.5 [1–7]	0.21
Mean arterial pressure (mmHg)	83 [77–93]	85 [77–93]	83 [76–94]	0.33
Mechanical ventilation [*n* (%)]	30 (56 %)	15 (57.7 %)	15 (53.6 %)	0.42
Weight’s variation (kg)	−2.5 [− 9.3; −2.3]	−6 [− 11; +1.5]	−1.5 [− 4.9; +4]	0.12
Prior RRT length (days)	11 [5–18]	10.5 [5–14.8]	11 [6.3–24.5]	0.46

Weight’s variation means between admission and D0

*p*: differences between S_+_ and S_−_ groups

*SOFA* Sequential Organ Failure Assessment

### Factors related to the success of weaning of RRT

Among the 54 patients, 26 attempts of weaning succeeded (S_+_ group) while 28 failed (S_−_ group). Baseline characteristics of the two groups were comparable (Table 1). The proportion of CVVHDF was more important in S_+_ group (50 vs 21 %) and conversely for IHDF, but differences were not statistically significant (*p* = 0.07). At the day of RRT cessation, the proportion of patients mechanically ventilated was similar between groups as were non-renal SOFA score and prior duration of RRT. Clinical parameters including mean arterial pressure, dose of vasopressors, mechanical ventilation and fluid balance did not differ between the two groups. However, a higher weight decrease was observed in the success group but without significant differences (Table 2).

**Fig. 2:**
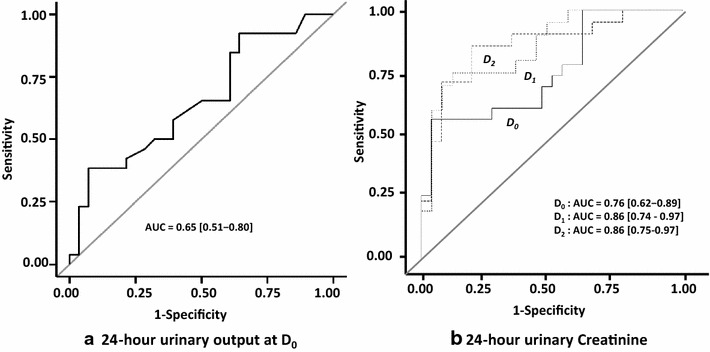
Impact of 24-h urine output (**a**) and 24-h urinary creatinine at Days 0, 1 and 2 (**b**) on predictive ability of successful discontinuation of renal replacement therapy. The area under the receiver operating characteristic curve (AUC) is shown in each graph

At D_0_, D_1_ and D_2_ urea and creatinine blood levels did not differ between the two groups (Table 3). At D_0_, 24-h urine output tended to be higher in S_+_ group (2300 vs 1950 ml, *p* = 0.052) but most of patients were treated by diuretics in both groups (73 vs 86 %). Diuretic response index was significantly higher in the S_+_ group at D_0_ with a significant decrease in furosemide dose the following two days as compared to S_−_ (Table 3). UrCl, CrCl, UUr and UCr levels were significantly higher in group S_+_ at D_0_, D_1_ and D_2_. UUr/UrGR and UCr/CrGR ratios were also significantly higher in S_+_ group (Table 3). By multivariate analysis, 24-h UCr was the most powerful variable to predict the success of RRT weaning. At D_0_, its area under the ROC curve (AUC and 95 % CI) was 0.76 [0.62–0.89] as compared to that of 24-h urine output: 0.66 [0.51–0.80] (Fig. 2). AUC of 24-h urinary creatinine was 0.86 [0.75–0.97] and 0.86 [0.75–0.97] at D_1_ and D_2_, respectively (Fig. 2). D_1_ 24-h creatininuria was a strong predictor of successful weaning with an odds ratio at 2.01 [1.27–3.18], *p* = 0.003, independently of age, weight and diuresis and showed Se, Sp, NPV and PPV at 75, 88, 82 and 84 %, respectively, when ≥5.2 mmol/24 h [3.1–6.3] (Table 4).

**Table 3:** Serum urea, serum creatinine, 24-h urine output, diuretics, urea and creatinine clearance, 24-h urinary urea and creatinine, urea and creatinine generation rate, urinary urea/urea generation rate and urinary creatinine/creatinine generation rate ratios at the day (D_0_) of attempt of weaning and the following two days (D_1_, D_2_)

	D0			D1			D2		
	S_+_	S_−_	*p*	S_+_	S_−_	*p*	S_+_	S_−_	*p*
S urea (mmol/l)	10 [7.6–13]	7.3 [6.2–12]	0.56	16 [12–24]	17 [14–21]	0.26	22 [15–37]	24 [20–27]	0.54
S creat (µmol/l)	105 [73–170]	83 [64–117]	0.36	177 [136–294]	158 [115–197]	0.15	227 [137–309]	218 [155–314]	0.65
24-h urinary output (l)	2.3 [1.5–3.5]	1.9 [1.0–2.5]	0.05	2.4 [1.8–3.7]	2.6 [1.7–3.3]	0.46	2.7 [2.1–3.4]	2.5 [1.7–3.3]	0.32
Use of furo [*n* (%)]	19 (73)	24 (86)	0.42	15 (59)	25 (89)	*0.009*	9 (35)	21 (75)	*0.005*
Dose of furo (mg/24 h)	470 [215–1000]	1000 [483–1000]	0.06	500 [125–970]	1000 [500–1000]	*0.01*	500 [250–1000]	1000 [500–1000]	*0.01*
Diuretic RI (ml/mg)	6 [2.9–15.4]	2.1 [1.1–6.4]	*0.02*	4.6 [3.6–26.4]	3.2 [1.9–4.3]	0.06	5.8 [2.5–8.2]	3.4 [2.6–5.8]	0.09
UrCl (ml/min)	15 [8–20]	6 [4–12]	*0.008*	12.5 [8–20]	7 [5–9]	*0.001*	13 [9–18]	6 [4–8]	<*0.001*
CrCl (ml/min)	31 [15–41]	18 [7–26]	*0.009*	31 [18–40]	15 [8–21]	*0.002*	31 [16–42]	13 [6–21]	<*0.001*
UUr (mmol/24 h)	187 [72–339]	85.9 [44–157]	*0.031*	357 [195–569]	147 [95–224]	*0.002*	592 [230–672]	236 [113–272]	*0.002*
UCr (mmol/24 h)	4.9 [2.5–6.4]	2.55 [0.7–3.9]	*0.002*	6.65 [5–8.8]	3.5 [1.6–4.4]	<*0.001*	8.1 [6–9.5]	4.4 [2.2–5.2]	<*0.001*
UrGR (µmol/min)				530 [331–722]	318 [254–442]	*0.02*	428 [257–681]	305 [232–393]	0.08
CrGR (µmol/min)				6.8 [5.8–9.9]	4.1 [2.9–5.2]	<*0.001*	6.2 [4.8–7.8]	4 [3.4–4.9]	*0.002*
UUr/UrGR (%)				51 [36–61]	36 [24–48]	*0.03*	71 [62–84]	48 [31–60]	<*0.001*
UCr/CrGR (%)				72 [53–85]	57 [40–67]	*0.047*	94 [77–100]	67 [44–82]	<*0.001*

Italic values indicate significance of *p* value (*p* <0.05)

Results are displayed in median [interquartile range] if quantitative variable; and number and percentage if categorial variable

*p*: differences between S_+_ and S_−_ groups

*S urea* serum urea, *S creat* serum creatinine, *Furo* furosemide, *Diuretic RI* diuretic response index, *Cl* clearance, *UUr* urinary urea, *UCr* urinary creatinine, *UrGR* urea generation rate, *CrGR* creatinine generation rate, *Ur* urea, *Cr* creatinine

**Table 4:** Performances of 24-h urine output, 24-h urinary creatinine and urea to predict the success of an attempt of renal replacement therapy weaning

	Youden index [95 % CI]	Sensitivity % [95 % CI]	Specificity % [95 % CI]	PPV % [95 % CI]	NPV % [95 % CI]
D0 urinary output (ml/24 h)	2575 [1050–3075]	38.5 [19–58]	93 [82–100]	85 [61.5–100]	62 [55–71]
D0 urinary urea (mmol/24 h)	134 [56–322]	65 [48–83]	73 [58–88.5]	68 [53–84]	70 [58–84]
D0 urinary creatinine (mmol/24 h)	4.7 [0.85–5.1]	56.5 [35–74]	96 [88.5–100]	93 [78–100]	71 [62.5–81]
D1 urinary urea (mmol/24 h)	293 [196–363]	70 [50–90]	88 [76–100]	83 [67–100]	79 [68–92]
D1 urinary creatinine (mmol/24 h)	5.15 [3.01–6.3]	75 [55–90]	88 [72–100]	84 [68–100]	82 [70–93]
D2 urinary urea (mmol/24 h)	318 [292–591]	71 [52–90]	85 [69–96]	80 [64–94]	79 [68–92]
D2 urinary creatinine (mmol/24 h)	5.56 [4.67–6.57]	86 [71–100]	81 [65–96]	78 [65–94]	88 [75–100]

*PPV* positive predictive value, *NPV* negative predictive value, *CI* confidence interval

### Patients’ outcome

A significantly shorter ICU length of stay (13.5 vs 24.5 days) and a shorter duration of RRT (10.5 vs 18 days) were observed in the S_+_ group (Table 1). Patients from the S_−_ group had resumed RRT 4 (3–11) days after the attempt of weaning. Twenty-one of the 28 patients of this group were weaned of RRT after a second attempt, whereas 7 needed more than two attempts (only one patient was re-dialyzed after the seventh day). Hospital survival was at 100 % in both groups, but 3 months later, 4 patients deceased in the S_−_ group and none in the S_+_ group.

## Discussion

In this study, we found that almost half of surviving AKI patients treated with RRT had their treatment stopped at the first attempt. We found also that the capability of 24-h urine output to predict RRT weaning was significantly altered by the use of diuretics. In contrast, 24-h urinary creatinine appeared as a powerful marker of successful discontinuation of RRT even if patients received diuretics.

A large majority of ICU patients with AKI may recover sufficient kidney function to be independent of RRT. Indeed, all surviving patients we investigated herein recovered and were weaned from RRT even if several weaning attempts were necessary in some of them. Currently, practices of RRT weaning during AKI depend on every center policy and vary from a “wait and see” and a “go fast” attitudes. The “wait and see” attitude may prolong needlessly RRT and expose the patient to its hazardous effects without any benefits. Conversely a “go fast” attitude may lead to several unsuccessful attempts of weaning with the subsequent requirement of re-institution that is by itself harmful. Appropriate cessation of RRT is obviously critical regarding clinical and economic outcomes. There is, however, a paucity of data about how and when to stop RRT in critically ill patients with AKI, and identification of predictive factors of successful cessation of RRT is scarce. This lack of evidence contrasts with the field of mechanical ventilation, where many studies dealing with the process of weaning have been conducted [30]. We sought therefore to evaluate, in the present study, whether cessation of RRT can be early determined on the basis of clinical and routinely used parameters.

Previous studies have reported the association of the success of an interruption of RRT with age [23], previous renal function [24, 25], the prior length of RRT, the SOFA score at ICU admission [23, 26] and its decrease from admission to the day of attempt [23]. None of these parameters have been found determinant in the success of weaning in our study. Blood parameters including serum urea and creatinine and their kinetic were also helpless to guide the weaning of RRT in our population. Urine output was reported to be the most important predictor of successful discontinuation of RRT. In the trial by Bouman et al. [31], continuous RRT was discontinued when the urine output returned to and was stable at more than 60 ml/h, but there were no data about the optimal rate of urine output predicting the success of weaning. Wu et al. [23] evaluated retrospectively 94 patients weaned from IHDF for 5 or more days, of which 64 were free from RRT for ≥30 days. 24-h diuresis was at 1435 ± 1172 versus 598 ± 700 ml in the success and the re-dialysis groups, respectively, but diuretics were used in 36 % of patients of both groups. Oliguria (100 ml in 8 h) was independently associated with re-initiation of RRT [23]. The BEST Kidney investigators [24] found that urine output of >400 ml/day without diuretics had the best operative characteristics with a Se, Sp, PPV and NPV of 0.47, 0.81, 0.81 and 0.77, respectively. However, diuretics administration negatively affected its predictive ability. They concluded that patients with more than 400 ml/day of urine without diuretics or more than 2300 ml/day with diuretics have more than 80 % of chance to successful discontinuation of RRT. Recently, an urine output >8.5 ml/kg/24 h was also found to predict IHDF weaning [32]. In our study, at the day of attempt, 24-h urine output failed to predict the success of weaning (2300 vs 1900 ml/24 h, *p* = 0.052) but a high proportion of our patients, more than 75 %, received furosemide. We observed, however, that furosemide induced a significantly higher diuresis in the success group. Indeed, recent studies reported a significantly higher urine output in case of successful discontinuation of RRT even if diuretics were used [25, 26]. Nevertheless, the usefulness of diuresis to guide RRT weaning is clearly reduced since diuretics often may be necessary to correct or avoid a fluid overload [33]. We sought then to determine predictive factors that would be independent of diuretics like urinary creatinine and urea. Urea and creatinine collected in urine during 24-h were significantly higher at the day of attempt of RRT weaning and the following 2 days (with a gradual increase) in the success group. By multivariate analysis, 24-h creatininuria was the most powerful predictor of success of weaning. A level ≥5.2 mmol/24 h had a Sp, Se, NPV and PPV of 75, 88, 82 and 84 %, respectively. Differences of creatinine urinary level observed between the two groups should not be related to muscle mass, as they were independent of patient’s age and weight. Also, these differences may not be explained by differences of urea or creatinine generation rate since ratios of urinary levels to generation rates were significantly higher in the success group. Of note, clearance of creatinine (and urea) was significantly higher in the S_+_ group, as reported by Fröhlich et al. [25]. In a recent study, Aniort and colleagues [32] sought also to identify predictive factors of intermittent hemodialysis weaning by investigating retrospectively 67 AKI patients. They found that daily urinary urea was the best weaning marker with an optimal threshold >1.35 mmol/kg/24 h (AUC 0.96, 95 % CI 0.93–0.99) as compared to 24-h urine output. In the contrary of our findings, they did not observe any differences in urinary creatinine between weaned and unweaned patients. However, almost 1/3 of their patients (22/67) have a baseline GFR < 30 ml/mn and expectedly 26/67 patients did not recovered renal function. Our population included exclusively patients with a baseline GFR ≥ 60 ml/mn explaining, at least for a part, these discordant results.

RRT modalities (continuous or intermittent) may affect renal recovery and dialysis dependence, but their effects remain a matter of debate. A systematic review of the literature including mainly observational studies concluded that patients who received intermittent RRT had a significant increased risk of dialysis dependency as compared to those who received continuous RRT [34]. We observed indeed a higher proportion of continuous therapies with a higher weight decrease in the success group. However, differences were not significant and our RRT strategy was based on hemodynamic precluding any conclusions from our study. It is noteworthy that two recent studies failed to demonstrate any impact of RRT modalities on renal recovery [35, 36].

Patients who require RRT for AKI have a mortality exceeding 60 %, but most patients that recover function enough to be free from RRT survive to hospital discharge [37]. In the study of BEST Kidney investigators [24], patients whose CRRT was successfully stopped had better outcome than those who needed to be re-treated (mortality: 28.5 vs 42.7 %, *p* = <0.0001, respectively). In our study, the patients who were weaned from RRT after a second or more attempt had a significantly higher length of RRT and of ICU stay as compared to patients successfully weaned. Moreover, we observed that 4/28 patients of the failed group deceased 3 months later and none in the success group. Whether failure of discontinuation of RRT is just a marker of severity of disease or is harmful by itself remains questioned [38].

This study has several limitations. First, it was a retrospective and bi-center cohort study. Second, the weaning of RRT was not based on standardized criteria but decided by the attending physician who obviously followed general rules. Third, daily urinary creatinine requires 24-h urine collection, a procedure routinely performed with accuracy in ICUs. The measurement of urinary creatinine in a shorter time period or as spot urine concentration would be more easily feasible but may induce less accuracy. Nevertheless, the use of urinary creatinine as a marker of RRT cessation should be internally and externally validated to be used in routine practice. Last, we defined the attempt of weaning as a discontinuation of RRT during 72 h and estimated a success if discontinuation lasted more than 15 days. Various definitions have been used including an interruption of RRT during at least 5 days [23], but we thought that this duration may be too short regarding the severity of our patients. Also, considering RRT weaned after 7 days of interruption, as chosen by others [24, 25] has revealed insufficient. In the BEST Kidney cohort, for example, 15 patients considered as RRT-free were re-treated by CRRT before discharged from the ICU [24]. Indeed, Wu et al. [23] observed that an average of 10.1 ± 6.1 days lasted before re-dialysis if the attempt of weaning RRT failed.

## Conclusions

In summary, the usefulness of 24-h urine output to predict the success of RRT weaning in AKI patients treated by diuretics may be significantly altered. Our study showed that 24-h urinary creatinine, in spite of diuretic use, is an independent and performing predictive factor of successful RRT weaning. A 24-h urinary creatinine ≥5.2 mmol indicates that RRT will be successfully stopped in 84 % of cases. Larger and prospective studies are needed to confirm our observations and to test a strategy of RRT weaning based on urine output, urinary creatinine and urea, and severity scores.

## Abbreviations

AKI: acute kidney injury
AUC: area under the curve
CI: confidence interval
CrCl: creatinine clearance
CrGR: creatinine generation rate
CVVHDF: continuous venovenous hemodiafiltration
D0, D1, D2: day of renal replacement therapy weaning attempt and the following 2 days
eGFR: estimated glomerular filtration rate
ICU: intensive care unit
IHDF: intermittent hemodiafiltration
NPV: negative predictive value
OR: odds ratio
PPV: positive predictive value
ROC: receiver operating characteristic
RRT: renal replacement therapy
SAPS: Simplified Acute Physiology Score
Se: sensitivity
SLEDD-f: sustained low-efficiency daily dialysis-filtration
SOFA: Sequential Organ Failure Assessment
Sp: specificity
UCr: 24-h diuresis urinary creatinine
UrCl: urea clearance
UrGR: urea generation rate
UUr: 24-h diuresis urinary urea
